# Unpacking socio-demographic predictors of child aggression: insights from a Saudi Arabian context from parents’ and caregivers’ perspectives

**DOI:** 10.3389/fpsyg.2025.1685361

**Published:** 2025-11-14

**Authors:** Ayoob Lone

**Affiliations:** Department of Clinical Neurosciences, College of Medicine, King Faisal University, Alhasa, Saudi Arabia

**Keywords:** socio-demographic factors, aggression, children, caregivers, parents, Saudi Arabia

## Abstract

Aggressive behavior in children is influenced by different sociodemographic factors. However, there is limited research on the impact of these factors on child aggression in Saudi Arabia. This study aimed to explore the sociodemographic factors associated with aggressive behavior among Saudi schoolchildren. A cross-sectional study was performed on 315 school-going children using stratified school selection with caregivers convenience sampling. Caregivers completed the Arabic caregivers-proxy version of the Buss Perry Aggression Questionnaire-Short form (BPAQ-SF). Multiple linear regression analysis was used to evaluate the association between various demographic variables and aggression. The significance level was set at a *p* < 0.05 throughout the analysis. The findings revealed a significant relationship between different sociodemographic variables and the BPAQ-SF. The results revealed that gender, age, family type, family occupation, parental education, and monthly income were found to be significant predictors of child aggression. Among these demographic factors, female gender (*β* = −0.15, *p* < 0.01), higher fathers (*β* = −0.19, *p* < 0.01), and mothers education (β = −0.47, *p* < 0.01) were associated with lower physical aggression scores. Verbal aggression was lower among children in nuclear families (*β* = 0.11, *p* < 0.05) and those with higher maternal education (*β* = −0.31, *p* < 0.01). Greater anger was observed among young children (<6 years; *β* = −0.24, *p* = 0.01), those with unemployed parents (*β* = −0.14, *p* = 0.01), lower paternal education (β = −0.19, *p* = 0.01), and maternal education (*β* = −0.29, *p* = 0.01), whereas extended family type showed a positive association with anger (*β* = 0.12, *p* = 0.05). Hostility was negatively related with maternal education (*β* = −0.25, *p* < 0.01) and monthly income (*β* = −0.15, *p* < 0.01). This study identified several demographic factors that played a role in the development of aggression in children. Thus, this study emphasizes that contributing factors should be considered when formulating and applying intervention strategies to manage aggressive behavior in children.

## Introduction

Aggressive behavior in school-going children is a pressing concern worldwide, with implications for academic performance, social relationships, and long-term mental health issues (Shanti and Moreno, 2023; Schwartz et al., 2015). Aggressive behavior is a major concern in growing children that needs to be resolved at its emergence in order to avoid further danger in later life. During the recent decade, aggressive behavior in children has drawn scientific attention due to its adverse consequences as well as the possibility of a high level of aggression, especially at an early age (Navarro et al., 2022; Pingault et al., 2013; Tremblay, 2010). Aggressive behaviors are intentional acts, such as hitting, kicking, biting, or pushing, that cause or threaten physical injury or psychological harm in interpersonal contexts (Ward et al., 2025; Hay et al., 2021; Eltink et al., 2018). According to Buss and Perry (1992), aggressive behavior can be exhibited in various forms, including physical aggression, verbal aggression, anger, and hostility. However, Little et al. (2003) argued that aggressive behavior is divided into three different categories, such as physical aggression, harming others using physical force (Norlander and Eckhardt, 2005); verbal aggression, hurting others by using words (Bodenmann et al., 2010); and relational aggression, breaking social relationships using manipulation (Crick and Grotpeter, 1995).

Several previous studies have examined the cognitive and emotional factors underlying the early formation of aggressive behaviors in children (Huitsing and Maks, 2018; O’Toole et al., 2017; Lee et al., 2016; Poland et al., 2016). A socioecological model suggested that there is a complex interplay between individual, relationship, community, and societal factors (Reupert, 2017). This helps to understand that many factors put people at risk for aggressive behavior or protect them from experiencing perpetrating aggression. Individual factors, such as biological and personal history (e.g., age, education, income, and substance abuse), could increase the chances of becoming a victim or perpetrator of aggression. Close relationships (peers, partners, and family members, etc.) may also increase the risk of aggression as a victim or perpetrator. Communities such as schools, workplaces, and neighborhoods where social relationships occur are associated with becoming victims or perpetrators of aggression. Social factors (norms, beliefs, economy, and inequality, etc.) help to create a climate in which aggressive behavior is encouraged or inhibited (Dahlberg and Krug, 2002). Among the social and physical environment, family variables are pivotal for the overall development of the child (Lin, 2023). Previous studies have reported that aggressive behavior is linked to many family factors (Navarro et al., 2022), such as single parents (Meysamie et al., 2013; Jansen et al., 2012; Baker et al., 2019), low socio-economic status (Meysamie et al., 2013; Jansen et al., 2012), low parental education background, (Meysamie et al., 2013; Jansen et al., 2012), unemployment (Jansen et al., 2012), working mothers (Kopp et al., 2024; Amin et al., 2011), parent–child conflict (Hammes et al., 2012; Ostrov and Bishop, 2008), physical abuse (Matheson et al., 2017), chronic disease and death of a family member (Meysamie et al., 2013), authoritarian and permissive parenting (Casas et al., 2006), hostile parents (Yoo and Ahn, 2023), working pattern of parent (Güngör et al., 2021), and mother’s severe negative emotional expressions (Mizokawa and Hamana, 2020).

Other sociodemographic variables such as gender and age have been found to influence the formation of aggression in school-going children. A significant number of previous studies have reported more aggressive behavior in males than females (He-Li et al., 2025; Koyama et al., 2024; Ibabe, et al., 2014; Contreras and Cano, 2014). However, mixed findings have been found regarding sex-related differences in aggression. Many researchers have reported higher rates in males than females for all types of aggression (Boxer, et al., 2009; Walsh and Krienert, 2007) but few studies have indicated that verbal aggression is more prevalent in females than in males, while physical aggression is more prevalent in males than in females (Antoñanzas et al., 2022; Jaureguizar et al., 2013; Calvete et al., 2013a). Regarding age, physical aggression was found in children in early childhood and increased by the age of 2 or 3 years (Alink et al., 2006). However, physical aggression in early childhood tends to reduce with age (Campbell et al., 2000; Ostrov et al., 2006), and peaked around 15 years of age (Karriker-Jaffe et al., 2008). Despite these established developmental patterns, there is a lack of empirical research in Saudi Arabia exploring how sociodemographic factors—such as gender, parental education, family structure, and socioeconomic status—interact to influence children’s aggressive behaviors. Given the nation’s unique cultural values, family systems, and parenting approaches, evidence derived from other populations may not accurately reflect the Saudi context. Most studies conducted in Western and neighboring Arab countries have explored overall aggression levels without differentiating between specific sub-dimensions such as physical aggression, verbal aggression, anger, and hostility (Solberg et al., 2025). Furthermore, few have examined these behaviors through the perspective of caregiver reports (Alabdulrazaq and Al-Haj Ali, 2020), which may provide valuable insight into the home environment where many aggressive behaviors are first observed. Therefore, the current study aims to fill this knowledge gap by identifying which sociodemographic variables are most strongly associated with each sub-dimension of aggression among Saudi school-aged children. By doing so, the study contributes context-specific evidence to inform early detection and targeted behavioral interventions. The present research is guided by the socio-ecological model (Reupert, 2017), which provides a comprehensive framework for understanding how aggression develops through interactions between individual, relational, and contextual factors. This model is particularly relevant to the Saudi setting, where children’s behavior is shaped by family systems, parental roles, and broader community influences. Applying this model allows for an integrated analysis of how sociodemographic characteristics—reflecting family and environmental contexts—collectively relate to aggressive behavior. Thus, the study not only extends existing literature but also situates child aggression within the cultural and social ecology of Saudi Arabia.

To the best of our knowledge, this is the only study to examine sociodemographic variables as predictors of aggression in school-going children. These variables were taken based on previous findings that indicate that these factors have a significant impact on the formation of aggressive behavior in children (Navarro et al., 2022). Therefore, the aim of the present study was to explore the role of sociodemographic factors in the emergence of aggressive behavior in school-going Saudi children.

## Materials and methods

### Study design

This study adopted a cross-sectional design to examine the impact of sociodemographic characteristics on the development of child aggression. Cross-sectional studies allow for the simultaneous collection of data on demographic factors (Kesmodel, 2018) and the Buss Perry Aggression Questionnaire-Short Form (BPAQ-SF) (Bryant and Smith, 2001) providing a snapshot of their relationships at a specific point in time. The Deanship of Scientific Research at King Faisal University in Al-Hasa, Saudi Arabia granted ethical approval for this study (KFU-REC-2023-OCT-ETHICS1574). The study was conducted in compliance with the Declaration of Helsinki on Research Involving Human Subjects. All participants and their parents or caregivers were fully informed about the study’s objectives and procedures. Written informed consent was obtained from parents and caregivers, and participation was entirely voluntary. Confidentiality and anonymity were strictly maintained in accordance with the principle of autonomy.

### Participants and sampling

The participants in this study were school-going children studying at different schools in the AlHasa Governorate of Saudi Arabia. A total of 340 children aged between 4 and 12 years were invited to participate in this study, and 315 participants completed the questionnaire, with a 92.65% response rate. Twenty-five participants were not included in the study due to missing data. The inclusion criterion was physically healthy children aged 4–12 years of age. Children above Grade 6, disabled children, non-Saudi individuals, and those who did not provide consent to participate were excluded from the study. Stratified random sampling was used to ensure that the sample accurately reflected the sociodemographic features of the population. When conducting research on the association between sociodemographic characteristics and child aggression, it is critical to include a wide range of demographic subgroups, such as school nature (private and governmental), student gender (male or female), geographical regions (eastern, western, southern, northern, and central), and area of residence (rural or urban). By separating the population into multiple strata based on these important criteria, stratified random sampling allowed for a more equal representation of each subgroup in the final sample. The population was first divided into different strata based on these factors, to ensure that each stratum was proportionally represented in the final sample. From each stratum, we selected schools purposively to ensure representation across these strata; within selected schools parents/caregivers were approached using convenience recruitment (face-to-face during school visits) and invited to participate. Thus, while schools were selected to achieve stratified coverage participant (caregivers) recruitment at each site was convenience-based. Because recruitment within sites was non-random, we frame our inferences as applicable to the sampled school/participants rather than the entire AlHasa population.

### Sample size calculation

For the present study, a total sample size of 340 was calculated using the following formula (*n* = *Z*^2^ × *p* × (1−*p*)/*E*^2^). This formula is mostly designed for stratified random sampling and a cross-sectional study design (Pourhoseingholi et al., 2013) that considers the prevalence of aggressive behavior in children in Saudi Arabia (Haddad et al., 2020; Alrokban et al., 2019) with a confidence interval of 95% and a 5% margin of error. Cases with missing responses on the BPAQ-SF or key sociodemographic variables were handled using list-wise deletion, ensuring that only complete cases (315) were included in the final analyses. The dataset was checked for data-entry errors, outliers, and inconsistencies prior to statistical testing.

### Data collection tools

#### Aggressiveness

Children’s aggressive behavior was assessed using the Buss Perry Aggression Questionnaire-Short Form (BPAQ-SF) (Bryant and Smith, 2001) of the Buss Perry Aggression Questionnaire (BPAQ) (Buss and Perry, 1992; Reyna et al., 2011). The BPAQ provides a standardized tool for assessing aggression, allowing for comparisons across research and populations. In clinical practice, the questionnaire can assist professionals in identifying individuals who may benefit from aggressive behavioral management strategies. Overall, the BPAQ is an important instrument for psychologists and academics, providing insights into the multifaceted nature of aggression and guiding efforts to reduce its detrimental effects on individuals and society (Gerevich et al., 2007). Originally BPAQ was designed for adolescents aged 18 + years; however, a short version of it has been applied and validated in many studies conducted among children (Torregrosa et al., 2020; Pechorro et al., 2016; Malaeb et al., 2020).

The BPAQ-SF consists of 12 items measuring four components of aggressive behavior: physical aggression, verbal aggression, anger, and hostility. Respondents were asked to rate each item on a 5-point Likert scale ranging from “1” (extremely uncharacteristic of me) to “5” (extremely characteristic of me). The total score was derived by adding the scores of items belonging to different areas, and the mean scores were obtained. A high score on this scale indicates high aggression. Buss and Perry (1992) reported the internal consistency reliability of the scales were 0.85 (Physical aggression), 0.72 (Verbal aggression), 0.83 (Anger), and 0.77 (Hostility). In the present sample, Cronbach’s alpha values indicated acceptable internal consistency for Physical Aggression (*α* = 0.81), Anger (*α* = 0.78), Hostility (*α* = 0.73), and marginal but adequate reliability for Verbal Aggression (*α* = 0.67). Exploratory factor analysis (principal component with varimax rotation) confirmed the four-factor structure, with all items loading on their expected subscales. Subscale inter-correlations (*r* = 0.41–0.58) and normal score distributions supported the scale’s construct validity for younger population.

#### Demographic questionnaire

The instrument covered demographic information, such as age, gender, and educational level. Furthermore, details regarding their families, such as living area, parental educational attainment, family type, income, occupation, and housing status were provided.

### Procedure

Trained senior medical students and interviewers conducted face-to-face interviews with parents or guardians of the selected children, ensuring confidentiality and cultural sensitivity. There is sometimes an implication of accurate information, since some parents have inadequate awareness about their children’s behavior or are hiding it, but it is still useful (Jokovic et al., 2004). Since it is culturally sensitive in Saudi Arabia to ask young children these types of questions directly, we used parents as a proxy for responding to the question. In addition to Saudi Arabia, the Western world is also concerned about this issue, as evidenced by the fact that one in four youngsters said that they were disturbed by survey questions about violence (Ybarra et al., 2009). In addition, collaboration with educational institutions has facilitated access to academic data. Parents or guardians gave informed consent to start the data collection process, emphasizing the voluntary nature of participation. Strict measures were taken to protect the privacy of participants, ensuring that the data were anonymized and securely stored. Prior to data collection, the questionnaire underwent a three-step translation and validation process. First, two bilingual professors fluent in English and Arabic translated the original questionnaire into Arabic, after which two other bilingual professors performed a back-translation into English. Second, expert reviewers evaluated the translated version and their feedback was incorporated to refine the questionnaire. Minor wording changes were applied to convert first-person items (e.g., I have threatened people I know) into caregiver-proxy wording (e.g., my child has threatened people he/she knows); changes preserved the original item intent. Finally, the Arabic version was pilot-tested on 25 healthy volunteers from the local community to evaluate its reliability and validity. Following this assessment, the expects approved the final version, which was then distributed through personal contacts. During interview training standardized probing and examples to ensure consistent responses. Interviews were conducted face-to-face by trained medical students; the responses were recorded as mothers, fathers, or other caregivers. Researcher selected caregiver proxy reports based on previous evidence demonstrating that parent proxy assessments provide acceptable reliability for evaluation externalizing behavior in young children (Jokovic et al., 2004). Additionally, the BPAQ-SF has been effectively adapted through proband-proxy pairs (Sanz-Gómez et al., 2025).

### Statistical analysis

Statistical Package for Social Sciences (SPSS) software (Version 27.0, Chicago, IL, USA) was used for statistical analysis. Descriptive statistics including frequencies, mean and standard deviation were used to characterize the study population and *p* value was considered statistically significant at *p* < 0.05 for the inferential analysis. Inferential statistics, such as the *t*-test and one-way analysis of variance, were applied to examine differences in socio-demographic variables and the BPAQ-SF. Independent-samples *t*-tests were conducted to examine differences in aggression subscales across the binary sociodemographic variables along with Cohen’s *d* (gender, family type, school type and housing status), and partial eta-squared (η^2^p) has been included for one-way ANOVA results examining differences across multiple sociodemographic categories (e.g., grades, family occupation, parental education and family income). These tests were appropriate as they enabled the identification of statistically significant differences between groups, helping to determine which demographic factors might be associated with aggression levels. Prior to regression modeling, the factor structure of the BPAQ-SF was verified using Exploratory Factor Analysis (EFA) conducted through Principal Axis Factoring (PAF) with Oblimin rotation (*δ* = 0) to allow for correlation between factors. Sampling adequacy was confirmed by Kaiser–Meyer–Olkin (KMO) = 0.82, Bartlett’s test of sphericity: *χ*^2^(66) = 812.37, *p* < 0.01 (confirming factorability), indicated that the data were suitable for factor analysis. The four-factor solution (physical aggression, verbal aggression, anger, and hostility) was retained, consistent with the theoretical structure of the BPAQ-SF, with all items loading on their expected factors (>0.45). Subsequently, multiple linear regression analysis were conducted separately for each of the four aggression subscales (physical aggression, verbal aggression, anger, and hostility) to identify socio-demographic predictors of each outcome. This test was selected because it identifies the strength and direction of the relationships between several independent socio-demographic variables and the dependent variable (aggression), allowing for a deeper understanding of which factors are significant (*p* < 0.05) predictors when controlling others. Before analysis, categorical variables were numerically coded as follows: Gender was coded as 0 = male and 1 = female. Parental education (few years of schooling = 1, primary = 2, high school = 3, graduate = 4, post graduate = 5), joint families (nuclear = 1, joint = 2) and monthly income (Saudi Riyals) (<5,000 = 1, 5,001–10,000 = 2, 10,001–15,000 = 3, >15,000 = 4) were treated as ordinal variables, where higher numeric values represented higher levels of education and income, respectively. Both unstandardized (*B*) and standardized (*β*) coefficients were reported, along with standard errors (SE), 95% confidence intervals (CI), and *p*-values. Model fit and effect sizes were expressed using the coefficient of determination (*R*^2^), adjusted *R*^2^, and Cohen’s *f*^2^ [computed as *R*^2^/(1 − *R*^2^)]. In addition, partial *R*^2^ values were calculated to estimate the unique variance explained by each predictor. To ensure that regression assumptions were met, several diagnostic procedures were conducted. Variance Inflation Factor (VIF) and tolerance values were computed to assess multicollinearity, with all predictors showing VIF < 4, indicating acceptable collinearity levels. Residual and influence diagnostics were examined, including standardized residuals, leverage values, and Cook’s distance. The Cook’s distance threshold was calculated as 4/*n* = 4/315 = 0.0127, and all cases fell below this value. Sensitivity analyses excluding borderline standardized residuals (|*z*| > 3) produced negligible changes in the regression coefficients, confirming the stability and robustness of the final models.

## Results

This study invited 340 school-going children studying in different schools in the Al-Hasa region of Saudi Arabia. A total of 315 students (147 males and 168 females) aged between 4 and 12 years completed the questionnaire. The remaining 25 participants who were reluctant to respond to all the questionnaire items were excluded. Table 1 shows that the majority (78.74%) of participants were enrolled in government schools. Only 67 participants (21.26%) were enrolled in private schools. Most of the participants (86; 27.30%) were studying in grade 6 and 25 (7.94%) were from kindergarten. The percentage of children belonging to nuclear and joint families was 79.05 and 20.95%, respectively.

**Table 1:** Buss Perry aggression questionnaire-short form (BPAQ-SF) scores according to demographic variables.

Variables	*N* (%) *n* (315)	Physical aggression (Mean and SD)	Verbal aggression (Mean and SD)	Anger (Mean and SD)	Hostility (Mean and SD)
Gender					
Male	147 (46.67)	**7.21** ± 3.05^**^	7.50 ± 2.63	7.50 ± 3.06	8.08 ± 2.50
Female	168 (53.33)	6.11 ± 2.87	7.43 ± 2.80	6.94 ± 3.16	7.98 ± 2.71
*t-test*		**3.26** ^**^	0.20	1.58	0.34
*Cohen’s d*		**0.37**	0.02	0.14	0.04
Age					
<6 Years	41 (13.02)	6.75 ± 2.93	7.92 ± 2.61	**7.93** ± 3.22^**^	7.92 ± 2.33
7–9 Years	121 (38.41)	6.86 ± 2.98	7.45 ± 2.54	7.70 ± 3.07	8.10 ± 2.76
>10 Years	153 (48.57)	6.40 ± 3.04	7.34 ± 2.87	6.62 ± 3.06	8.00 ± 2.58
***ANOVA***		1.89	1.69	**2.67** ^**^	2.16
***Partial η*** ^***2***^		0.02	0.02	**0.03**	0.02
School type					
Government	248 (78.74)	6.47 ± 2.95	7.34 ± 2.71	7.05 ± 3.19	7.95 ± 2.64
Private	67 (21.26)	**7.17** ± 3.12^*^	7.89 ± 2.70	**7.77** ± 2.83^*^	8.23 ± 2.51
*t-test*		**−1.79** ^*^	−1.47	**−1.69** ^*^	−1.03
*Cohen’s d*		**0.22**	0.13	**0.20**	0.11
Educational level					
Kindergarten	25 (7.94)	6.44 ± 2.88	7.56 ± 2.39	7.24 ± 3.07	7.60 ± 2.06
Grade 1	60 (19.05)	7.10 ± 3.24	7.60 ± 2.73	7.80 ± 3.30	8.06 ± 2.71
Grade 2	32 (10.16)	6.65 ± 2.58	7.93 ± 2.46	7.62 ± 2.79	8.09 ± 2.31
Grade 3	31 (9.84)	7.41 ± 3.06	7.90 ± 2.42	7.83 ± 2.73	8.61 ± 2.82
Grade 4	36 (11.43)	6.52 ± 3.01	6.58 ± 2.90	6.41 ± 3.04	7.50 ± 2.68
Grade 5	45 (14.28)	5.80 ± 2.93	7.20 ± 2.98	6.37 ± 3.45	7.97 ± 3.01
Grade 6	86 (27.30)	6.53 ± 2.98	7.51 ± 2.74	7.16 ± 3.06	8.16 ± 2.49
***ANOVA***		1.45	1.05	1.06	1.10
***Partial η*** ^***2***^		0.01	0.01	0.01	0.01
Family type					
Nuclear	249 (79.05)	6.45 ± 2.97	7.27 ± 2.64	6.95 ± 3.02	8.00 ± 2.64
Joint	66 (20.95)	**7.27** ± 3.03^*^	**8.18** ± 2.89^*^	**8.15** ± 3.34^**^	8.15 ± 2.53
*t-test*		**−1.97** ^*^	**−2.43** ^*^	**−2.78** ^**^	−1.89
*Cohen’s d*		**0.23**	**0.28**	**0.32**	0.21
Area of residence					
Urban	285 (90.48)	6.52 ± 2.91	7.39 ± 2.66	7.12 ± 3.11	7.95 ± 2.60
Rural	30 (9.52)	7.56 ± 3.65	8.10 ± 3.19	7.96 ± 3.24	8.83 ± 2.66
*t-test*		−1.08	−1.35	−1.40	−1.56
*Cohen’s d*		0.11	0.13	0.13	0.14
Family occupation					
Government job	219 (69.52)	6.66 ± 2.95	7.48 ± 2.68	7.30 ± 3.11	8.08 ± 2.60
Private job	68 (21.59)	5.95 ± 2.73	7.20 ± 2.65	6.80 ± 3.11	7.39 ± 2.59
Business	20 (6.35)	7.60 ± 3.97	7.60 ± 3.40	6.60 ± 3.42	**9.40** ± 2.54^**^
Unemployed	8 (2.54)	**8.87** ± 2.41^**^	8.62 ± 2.38	9.50 ± 1.85	8.75 ± 1.90
***ANOVA***		**1.73** ^**^	0.54	0.49	**1.61** ^**^
***Partial η*** ^***2***^		**0.02**	0.01	0.01	**0.02**
Fathers education					
Few years of schooling	13 (4.13)	**10.69** ± 2.25^**^	**9.76** ± 1.83^**^	**10.15** ± 2.54*^*^	9.46 ± 2.06
Primary	25 (7.94)	9.28 ± 3.66	9.40 ± 2.92	9.48 ± 3.36	**10.16** ± 3.14^**^
High school	50 (15.87)	7.16 ± 3.02	7.74 ± 2.79	8.14 ± 3.42	8.40 ± 2.44
Graduate	204 (64.76)	6.02 ± 2.60	7.00 ± 2.49	6.59 ± 2.77	7.64 ± 2.42
Post graduate	23 (7.30)	5.65 ± 2.38	7.47 ± 3.13	6.47 ± 3.13	7.60 ± 2.96
***ANOVA***		**8.23** ^**^	**4.16** ^**^	**3.74** ^**^	**2.17** ^**^
***Partial η*** ^***2***^		**0.11**	**0.04**	**0.03**	**0.02**
Mothers education					
Few years of schooling	23 (7.30)	**9.69** ± 3.13^**^	**9.65** ± 2.49*^*^	**9.52** ± 3.10^**^	**9.73** ± 2.28^**^
Primary	69 (21.90)	8.91 ± 2.85	8.49 ± 2.62	8.62 ± 2.77	9.15 ± 2.74
High school	46 (14.61)	6.63 ± 2.84	7.73 ± 2.76	7.56 ± 3.23	8.02 ± 2.26
Graduate	162 (51.43)	5.36 ± 2.12	6.77 ± 2.47	6.29 ± 2.80	7.41 ± 2.44
Post graduate	15 (4.76)	5.06 ± 2.60	5.93 ± 2.49	5.86 ± 3.50	6.93 ± 2.65
***ANOVA***		**14.97** ^**^	**4.73** ^**^	**5.93** ^**^	**3.78** ^**^
***Partial η*** ^***2***^		**0.18**	**0.06**	**0.08**	**0.03**
Monthly income (Saudi Riyals)					
<5,000	37 (11.75)	6.78 ± 3.33	8.01 ± 3.08	**8.05** ± 3.97^*^	**8.89** ± 3.08^**^
5,001–10,000	80 (25.40)	7.23 ± 2.94	7.71 ± 2.52	7.81 ± 3.06	8.36 ± 2.56
10,001–15,000	107 (33.96)	6.09 ± 2.81	7.26 ± 2.55	6.76 ± 2.71	7.72 ± 2.28
>15,001	91 (28.89)	6.65 ± 3.06	7.21 ± 2.88	6.84 ± 3.15	7.75 ± 2.75
***ANOVA***		0.81	1.30	**1.39** ^*^	**1.14** ^**^
***Partial η*** ^***2***^		0.01	0.01	**0.01**	**0.01**
Housing status					
Rented	98 (31.11)	6.77 ± 2.99	7.62 ± 2.67	7.26 ± 2.80	7.91 ± 2.36
Own	217 (68.89)	6.56 ± 3.01	7.39 ± 2.74	7.17 ± 3.27	8.08 ± 2.72
*t-test*		0.58	0.70	0.22	−0.53
*Cohen’s d*		06	0.8	0.3	0.5

Bold font = ^*^p < 0.05; ^**^p < 0.01.

Independent-samples *t*-tests were conducted to examine gender differences across the four subscales of the Buss–Perry Aggression Questionnaire–Short Form (BPAQ-SF). Boys scored significantly higher than girls on physical aggression [*t* (1, 313) = 3.26, *p* = 0.02, Cohen’s *d* = 0.37], indicating a small-to-moderate effect size where boys exhibited greater physical aggression than girls. No meaningful gender differences were observed for verbal aggression [*t* (1, 313) = 0.20, *p* = 0.84, *d* = 0.02], anger [*t* (1, 313) = 1.58, *p* = 0.14, *d* = 0.18], or hostility [*t* (1, 313) = 0.34, *p* = 0.73, *d* = 0.04]. In addition to gender, *t*-tests were performed for other binary socio-demographic variables. Children from private schools reported higher score on physical [*t* (1, 313) = 1.79, *p* = 0.03, *d* = 0.22], and anger [*t* (1, 313) = 1.69, *p* = 0.04, *d* = 0.20], subscale in comparison to children studying in government schools indicating small to moderate effect size.

Similarly, children from joint families scored higher on physical [*t* (1, 313) = 1.97, *p* = 0.05, *d* = 0.23], verbal [*t* (313) = 2.43, *p* = 0.02, *d* = 0.28], and anger [*t* (1, 313) = 2.78, *p* = 0.01, *d* = 0.32] subscales compared with those from nuclear families, indicating small-to-moderate effects. No statistically significant or practically meaningful differences were observed by area of residence and housing status (all *p* > 0.05, *d* < 0.15). These findings suggest that residential areas and housing status modestly contributes to variations in aggression levels among children.

One-way ANOVAs were conducted to examine the influence of age, educational level, parental education, family occupation, and income on the four aggression subscales. Physical aggression differed significantly by father’s education [*F* (4, 310) = 8.23, *p* < 0.01, η^2^ₚ = 0.11] and mother’s education [*F* (4, 310) = 14.97, *p* < 0.01, η^2^ₚ = 0.18], indicating large effects of parental education on this subscale. Family occupation [*F* (4, 310) = 1.73, *p* = 0.04, η^2^ₚ = 0.02] also showed a small to medium but significant effect, with children of unemployed parents scoring higher than those from children with employed parents.

For verbal aggression, significant differences were found for father’s education [*F* (4, 310) = 4.16, *p* < 0.01, η^2^ₚ = 0.04] and mother’s education [*F* (4, 310) = 4.73, *p* < 0.01, η^2^ₚ = 0.06], representing medium effects.

Regarding anger, significant differences were observed for age [*F* (3, 311) = 2.67*, p* < 0.01, η^2^ₚ = 0.03] represents small but significant effect. Father’s education [*F* (4, 310) = 3, 74, *p* < 0.01, η^2^ₚ = 0.03] and mother’s education [*F* (4, 310) = 5.93, *p* < 0.01, η^2^ₚ = 0.08], suggesting medium-to-large effects. Monthly income [*F* (4, 310) = 1.39, *p* = 0.05, η^2^ₚ = 0.01] showed smaller but statistically significant effects, with lower-income families tending to report slightly higher anger scores.

For hostility, significant differences were noted for family occupation [*F* (4, 310) = 1.61, *p* < 0.01, η^2^ₚ = 0.02] depicts small effects. Also, father’s education [*F* (4, 310) = 2.17, *p* < 0.01, η^2^ₚ = 0.02] and mother’s education [*F* (4, 310) = 3.78, *p* < 0.01, η^2^ₚ = 0.03], reflecting small effects. Similarly, monthly income [*F* (4, 310) = 1.14, *p* = 0.01, η^2^ₚ = 0.01] showed smaller but statistically significant effects, with lower-income families tending to report slightly higher anger scores. No significant differences were detected across education level (all *p* > 0.05), suggesting that education status had minimal influence on children’s aggression levels.

A separate multiple linear regression analysis was performed to identify predictor variables for physical aggression, verbal aggression, anger, and hostility, as measured using the BPAQ-SF. The results of the multiple regression analysis presented in Table 2 revealed a significant contribution of 11 predictor variables (gender, age, school type, education level, family type, and parental education, etc.) to explaining the scores on physical aggression, *R* = 0.61, *R*^2^ = 0.37, *F* (11, 303) = 16.31, *p* < 0.01. These variables jointly explained 37% of the variance in the physical aggression scores. Regression coefficients indicated that sex (male = 0, female = 1) was negatively and significantly related to physical aggression (*β* = −0.15, *p* < 0.01). This means that male participants were more aggressive than female participants in terms of the physical aspects of the BPAQ-SF. The results also revealed that fathers’ educational background (few years of schooling = 1, primary = 2, high school = 3, graduate = 4, postgraduate = 5) was negatively and significantly (*β* = −0.19, *p* < 0.01) associated with physical aggression. This indicates that children with a low level of father’s education had more physical aggression than children with a good father’s educational background. Similarly, the mothers’ educational level was negatively and significantly related to physical aggression (*β* = −0.47, *p* < 0.01). This means that children with a poor mother’s educational level had more physical aggression than did children with a good mother’s educational background.

**Table 2:** Result of multiple regression analysis predicting aggression using BPAQ-SF from demographic variables (*N* = 315).

Predictor	*R*	*R* ^2^	*F* (11, 303)	Unstandardized coefficient		Standardized coefficient	Level of significance	95% CI	
				*B*	*SE*	*β*		Lower	Upper
Physical aggression									
Gender	0.61	0.37	16.31 *p* < 0.01	−0.90	0.28	−0.15	0.00	−1.45	−0.35
Age				0.08	0.33	0.20	0.79	−0.56	0.73
School type				0.31	0.36	0.04	0.38	−0.39	1.02
Educational level				−0.12	0.11	−0.08	0.27	−0.33	0.09
Family type				0.60	0.35	0.08	0.08	−0.08	1.29
Area of residence				0.36	0.47	0.04	0.44	−0.57	1.30
Family occupation				−0.32	0.20	0.08	0.11	−0.71	0.08
Fathers education				−0.64	0.18	−0.19	0.00	−0.99	−0.28
Mothers education				−1.30	0.15	−0.47	0.00	−1.60	1.00
Monthly income				0.13	0.15	0.04	0.37	−0.16	0.42
Housing status				−0.27	0.30	−0.04	0.38	−0.86	0.33
Verbal aggression									
Gender	0.41	0.17	5.63, *p* < 0.001	0.06	0.29	0.01	0.84	−0.51	0.63
Age				−0.20	0.34	−0.05	0.56	−0.87	0 0.48
School type				0.47	0.37	0.07	0.20	−0.26	1.20
Educational level				0.01	0.11	0.01	0.90	−0.21	0.24
Family type				0.76	0.36	0.11	0.04	0.05	1.47
Area of residence				0.16	0.49	0.02	0.74	−0.81	1.14
Family occupation				−0.33	0.21	−0.09	0.11	−0.75	0.08
Fathers education				−0.32	0.19	−0.10	0.08	−0.70	0.04
Mothers education				−0.79	0.16	−0.31	0.00	−1.11	−0.48
Monthly income				−0.11	0.15	−0.04	0.48	−0.41	0.19
Housing status				−0.27	0.32	−0.04	0.39	−0.89	0.35
Anger									
Gender	0.49	0.24	8.87, *p* < 0.001	−0.46	0.32	−0.07	0.15	−1.09	0.17
Age				−1.07	0.38	−0.24	0.01	−0.1.82	−0.33
School type				0.48	0.41	0.06	0.24	−0.32	1.29
Educational level				0.16	0.13	0.11	0.19	−0.08	0.41
Family type				0.90	0.40	0.12	0.02	0.12	1.68
Area of residence				−0.01	0.54	0.00	0.99	−1.07	1.06
Family occupation				−0.59	0.23	−0.14	0.01	−1.04	−0.13
Fathers education				−0.65	0.21	−0.19	0.00	−1.06	−0.24
Mothers education				−0.84	0.17	−0.29	0.00	−1.18	−0.50
Monthly income				−0.29	0.17	−0.09	0.09	−0.62	0.05
Housing status				−0.13	0.35	−0.02	0.71	−0.81	0.55
Hostility									
Gender	0.37	0.14	4.31, *p* < 0.001	−0.04	0.29	−0.01	0.89	−0.61	0.53
Age				−0.11	0.34	−0.03	0.75	−0.77	0.56
School type				0.55	0.37	0.09	0.14	−0.17	1.26
Educational level				0.05	0.11	0.04	0.63	−0.17	0.27
Family type				−0.04	0.35	−0.01	0.92	−0.73	0.66
Area of residence				0.55	0.49	0.06	0.26	−0.41	1.51
Family occupation				−0.16	0.21	−0.05	0.44	0.57	0.24
Fathers education				−0.34	0.18	−0.12	0.06	−0.71	0.02
Mothers education				−0.61	0.16	−0.25	0.00	−0.92	−0.31
Monthly income				−0.43	0.17	−0.15	0.04	−0.53	0.07
Housing status				0.14	0.31	0.03	0.65	−0.47	0.75

Gender coded as 0 = male and 1 = female. Parental education (few years of schooling = 1, primary = 2, high school = 3, graduate = 4, post graduate = 5), joint families (nuclear = 1, joint = 2) and monthly income (Saudi Riyals) (<5,000 = 1, 5,001–10,000 = 2, 10,001–15,000 = 3, >15,000 = 4), where higher numeric values represented higher levels. *B* = unstandardized coefficient; *β* = standardized coefficient; *SE* = standard error; CI = confidence interval; *p* < 0.05 was considered statistically significant.

Demographic variables were entered into multiple regression analysis to examine their role in predicting verbal aggression. In this analysis (Table 2), family type was a significant predictor of verbal aggression (*β* = 0.11, *p* < 0.05). This implies that children belonging to joint families (nuclear = 1, joint = 2) experienced more verbal aggression than did children living in nuclear families. Mothers’ educational level was negatively and significantly related to verbal aggression (*β* = −0.31, *p* < 0.01) related with verbal aggression. This shows that children with a low level of mother’s education had more verbal aggression than children with a good mother’s educational background. None of the other demographic variables were found to be significant predictors of verbal aggression. However, all variables jointly accounted for 17% of the variance in verbal aggression, *R* = 0.41, *R*^2^ = 0.17, *F* (11, 303) = 5.63, *p* < 0.01, which was statistically significant.

Age, family occupation, family type, and parental education were significant predictors of anger. Age was negatively and significantly related to anger (*β* = −0.24, *p* < 0.01). This shows that children below 6 years of age expressed more anger than older children. Family occupation was also negatively and significantly correlated with anger (*β* = −0.14, *p* < 0.01). This indicates that children with unemployed parents had more anger than children who parents with jobs. The results also showed that fathers’ education level (few years of schooling = 1, primary = 2, high school = 3, graduate = 4, postgraduate = 5) was negatively and significantly (*β* = −0.19, *p* < 0.01) associated with anger. This indicates that children with a low level of father’s education had more anger than children with a good father’s educational background. Similarly, the mothers’ educational level was negatively and significantly related to anger (*β* = −0.29, *p* < 0.01). This means that children with a poor mother’s educational level had more anger than did children with a good mother’s educational background. However, family type (*β* = 0.12, *p* < 0.05) was a significant predictor of anger among children. The results clearly revealed that participants belonging to joint families showed more anger than those living in nuclear families did. Other demographic variables, such as gender, school type, educational level, area of residence, monthly income, and housing status, were not found to be significant predictors of anger. However, all the variables jointly accounted for 24% of the variance in the anger scores [*R* = 0.49, *R*^2^ = 0.24, *F* (11, 303) = 8.87, *p* < 0.01], which was significant.

While predicting hostility from different demographic factors, mother’s education level was found to be a significant predictor of hostility (*β* = −0.25, *p* < 0.01). This means that children with poor mothers’ educational level had more hostility than children with a high level of mothers’ education. Monthly income was negatively and significantly related to hostility (*β* = −0.15, *p* < 0.01). Children with low socioeconomic status showed more hostility than those with a high standard of life. None of the other demographic variables were found to be significant predictors of hostility. However, all variables jointly accounted for 14% of the variance in hostility scores [*R* = 0.37, *R*^2^ = 0.14, *F* (11, 303) = 4.31, *p* < 0 0.01], which was significant.

## Discussion

This is the first research of its kind to examine the impact of socio-demographic characteristic on child aggression in Saudi Arabia. This study revealed a substantial association between sociodemographic characteristics and the development of aggression among school-going Saudi children. The results indicated that gender, age, school type, family type, family occupation, parental education, and monthly income were significant predictors of child aggression, as reported by parents or caregivers.

The findings of the present study indicate significant differences between the mean scores of the BPAQ-SF and gender. The results showed that male participants scored higher on physical aggression than did female participants. Statistically significant differences between male and female participants in terms of physical aggression measured by the BPAQ-SF have been confirmed in several studies (Guo, 2025; John et al., 2023; Antoñanzas et al., 2022; Suárez-Relinque et al., 2019; Jaureguizar et al., 2013; Reyna et al., 2011; Calvete et al., 2013b; Pagan et al., 2009; Ulman and Strauss, 2003). The difference in aggressive behavior has been attributed to higher testosterone levels in males (Björkqvist, 2018; Archer, 2009; Archer, 2006). According to social role theorists, men are more likely to be physically aggressive because of their dominant and competitive roles in society (Hoff et al., 2009). Physical aggression is used by males to achieve social dominance and effectively compete for status and resources, such as access to relationships, peer networks, and popularity (Evans et al., 2019).

Regarding age, a significant difference was observed in the anger domain of the aggression scale. As depicted in the results, children below the age of 6 years scored higher in the anger domain of the BPAQ-SF in comparison to other age groups. This result supports previous research indicating that aggressive behaviors decline with age (Lee and Choi, 2025), largely due to cognitive maturation and enhanced socialization (Baker et al., 2019; Duggins et al., 2016). It is also consistent with neuroscience-based studies linking aggression reduction to brain development. As children grow older, structural and functional changes in the amygdala and hippocampus contribute to better emotional and behavioral regulation (Bos et al., 2018; Roberts et al., 2021). The amygdala is central to emotional processing and threat response, while the hippocampus facilitates memory formation, cognitive processing, and learning from experience—all of which play crucial roles in moderating aggressive impulses and negative reactions.

Interestingly, a significant difference was observed between the school type and aggression. Our results showed that participants enrolled in private schools scored higher in physical aggression and anger as compared to children studying in government schools. Children from higher-income families prefer private schools to experience different socialization processes than children from lower-income families in government schools. Peer influence may also be another factor that should be considered. Children in private schools may be exposed to different social dynamics and peer groups that can influence their behavior. Vitaro et al. (2002) believed that peer relationships have an impact on aggression levels in school settings. Moreover, academic pressure and competition in private schools may contribute to higher levels of stress and tension among children, potentially leading to increased aggressive behavior. It is important to note that aggression levels can vary widely within both private and government school settings, and individual differences play a significant role in shaping behavior. Factors such as parenting style, personal experience, and school climate also influenced aggression levels among children.

Regarding family type, participants from the joint family scored higher in the physical, verbal, and anger domains of aggression than participants living in nuclear families. These results are inconsistent with those of previous studies (Khan et al., 2014). The relationship between family types and aggression is complex. While there is no consensus, several factors may influence aggression levels in children from joint and nuclear families. In joint families, children may be exposed to multiple caregiving adults, including grandparents, uncles, aunts, and cousins. This extended support network can provide children with diverse social interactions and emotional support, potentially fostering a sense of security and stability, which can reduce aggression. Conversely, conflicts and disengagement among family members in joint families could lead to increased stress and tension within the household, which may contribute to higher levels of aggression among children.

Previous studies examined the relationship between occupation and child aggression. Most studies have reported a positive correlation with perpetration. The children of working mothers showed more aggressive behavior than those of unemployed mothers (Meysamie et al., 2013; Amin et al., 2011). However, our results indicated that children with unemployed parents had higher scores in the anger domain of aggression than children with employed parents. The children of unemployed parents may exhibit higher levels of aggression due to various interconnected factors. Economic stress and instability resulting from unemployment can lead to increased family conflicts, creating a hostile and chaotic home environment. This environment can contribute to higher levels of aggression in children as they may lack sufficient emotional support and structure. Moreover, the lack of resources and opportunities linked to parental unemployment, such as unstable housing, inadequate nutrition, and limited access to quality education and healthcare, can contribute to increased aggression among children. These external stressors can affect children’s overall well-being and increase their likelihood of behavioral problems, including aggression.

Another significant factor found in this study was that children with low paternal education levels showed higher scores for the physical aggression and anger domains of aggression. These results are consistent with those of previous studies, which reported that low parental education aggravates the risk of children being perpetrators or victims (Jansen et al., 2012). Jia et al. (2014) reported that children with low parental educational attainment exhibited both proactive and reactive aggressive behaviors. However, the study by Zhou et al. (2017) demonstrated through a moderated mediation model that lower levels of parental education were linked to increased aggressive behavior. In our study, maternal education was associated with child aggression. Our results clearly revealed that children with low maternal educational background showed more aggression in terms of physical aggression, verbal aggression, anger, and hostility. These findings are in line with those of previous studies, which found that maternal educational attainment below the undergraduate level was associated with parent-reported verbal aggression (Baker et al., 2020). Children of parents with lower education levels may be more likely to exhibit aggression due to various interconnected factors. One reason for this association is the effect of low parental education levels on parenting practices and family dynamics. Parents with lower educational attainment may face challenges in providing adequate emotional support, setting boundaries, and managing their children’s discipline effectively. Moreover, the children of parents with low educational levels may also face challenges in school, such as academic difficulties and social exclusion, which can further contribute to feelings of frustration and aggression.

The analysis also showed that hostility was statistically higher in participants with a low monthly income (<5,000 SAR) compared those with a high monthly income. Our findings echoed the results of previous studies (Jansen et al., 2012) which reported that children with poor socioeconomic conditions had a higher risk for all types of aggression. Another study found that children with low socioeconomic status scored higher for relational aggression, but not for physical aggression, than children with high socioeconomic status (Baker et al., 2020).

The findings of this study carry significant implications for parents, educators, and policymakers aiming to reduce aggressive behaviors among children. As maternal and paternal education emerged as the most influential protective factors, targeted interventions should emphasize strengthening parental awareness, communication, and emotional regulation skills. Community-based parenting programs and workshops could equip parents with effective strategies for positive discipline, emotional management, and constructive family communication—key components that have been shown to decrease aggression in children. Within the educational setting, teachers and school counselors play an equally vital role. Incorporating social and emotional learning into school curricula can help students identify and manage emotions, build empathy, and enhance problem-solving and interpersonal skills. Early identification of aggressive behaviors and the implementation of supportive interventions, such as behavioral counseling and peer mediation, can further reinforce these competencies in the classroom. At the policy level, collaboration between educational and public health institutions is essential. Developing parent education and family engagement programs—especially for parents with lower educational attainment—can provide accessible tools for managing children’s emotional and behavioral challenges. Partnerships between schools and community health centers can also facilitate workshops and consultation sessions that help parents reduce stress, improve parent–child relationships, and create emotionally supportive home environments. Overall, these findings underscore that empowering parents and educators through structured education and training initiatives can substantially mitigate aggressive tendencies and foster children’s emotional and social development, both within Saudi Arabia and in comparable cultural contexts.

Similar to other studies, our study had certain limitations. First, the study’s cross-sectional design limits its ability to establish causal relationships between demographic characteristics and aggressive behavior. Second, the findings of this study may not be generalizable beyond the specific context of the eastern governorate of Saudi Arabia. Cultural, social, and economic factors unique to this region may have influenced the results, limiting their applicability to other populations. In addition, this study could be more interesting for children from other regions of Saudi Arabia to analyze whether there are sociocultural differences. Third, the inclusion criterion of physically healthy children may have introduced a degree of selection bias and further limited generalizability. Some physical or chronic health conditions are known to be associated with emotional and behavioral difficulties; however, this criterion was applied to ensure that the aggression outcomes measured were not confounded by medical or neurological disorders. Fourth, the use of self-reported measures relies on parents/guardians reporting on behalf of children, which can introduce bias (Jokovic et al., 2004). Longitudinal studies would provide a more robust understanding of temporal dynamics and potential causal pathways. Finally, a quantitative design was chosen for this study, although a qualitative design might yield innovative information about the formation of aggressive behavior exhibited by this innocent population. Future research using a qualitative design could focus on uncovering the true antecedents that play a specific role in developing aggressive behavior in these children.

## Conclusion

This study examined the sociodemographic predictors of aggressive behaviors in Saudi Arabian school-going children from the perspectives of parents and caregivers. The findings highlighted significant associations between child aggression and several sociodemographic factors, including gender, age, school type, family type, parental occupation, parental education, and monthly income. These insights contribute to a deeper understanding of the factors that influence aggression among school-aged children in Saudi Arabia. Based on these findings, a comprehensive intervention strategy that includes parents and teachers can be developed to address and reduce aggressive behavior among children. Engaging parents and educators in these intervention programs may enhance support for children struggling with aggression and foster a more positive developmental environment.

## Data Availability

The original contributions presented in the study are included in the article/Supplementary material, further inquiries can be directed to the corresponding author.
