# Supplementary material for: Unpacking socio-demographic predictors of child aggression: insights from a Saudi Arabian context from parents’ and caregivers’ perspectives
Source: Front Psychol. 2025 Nov 14;16:1685361. doi: 10.3389/fpsyg.2025.1685361 (PMC12662091; doi:10.3389/fpsyg.2025.1685361)
Supplement: Supplementary file 1 [file Table_1.DOCX]

**Table S1: Item-level diagnostics: means, SD, item–total correlations, α-if-item-deleted, subscale α.**

| **Item** | **Subscale** | **Mean** | **SD** | **Item–Total correlation (within subscale)** | **α-if-item-deleted** | **Subscale α** |
| --- | --- | --- | --- | --- | --- | --- |
| Q1 | Physical | 2.46 | 1.24 | 0.89 | 0.23 | 0.81 |
| Q5 | Physical | 2.77 | 1.26 | 1.08 | 0.13 | 0.81 |
| Q9 | Physical | 2.36 | 1.30 | 0.96 | 0.15 | 0.81 |
| Q2 | Verbal | 2.36 | 1.28 | 0.54 | 0.51 | 0.78 |
| Q6 | Verbal | 2.61 | 1.21 | 0.56 | 0.50 | 0.78 |
| Q10 | Verbal | 2.37 | 1.18 | 0.55 | 0.51 | 0.78 |
| Q3 | Anger | 2.65 | 1.08 | 0.11 | 0.45 | 0.67 |
| Q7 | Anger | 1.91 | 1.14 | 0.10 | 0.50 | 0.67 |
| Q11 | Anger | 2.16 | 1.13 | 0.29 | 0.39 | 0.67 |
| Q4 | Hostility | 2.35 | 1.24 | 0.31 | 0.52 | 0.73 |
| Q8 | Hostility | 2.69 | 1.17 | 0.24 | 0.61 | 0.73 |
| Q12 | Hostility | 2.61 | 1.25 | 0.22 | 0.62 | 0.73 |

**Note**: Item–Total correlation = correlation of the item with the sum of the other items in the same subscale.

α-if-item-deleted shows the alpha of the remaining two items (useful for 3-item subscales).

Subscale α values reported in rightmost column.

**Table S2: Item descriptive (Q1–Q12): (Mean, SD, skewness, kurtosis)**

| **Item** | **Mean** | **SD** | **Skew** | **Kurtosis** |
| --- | --- | --- | --- | --- |
| Q1 | 2.46 | 1.24 | 0.51 | -0.90 |
| Q2 | 2.36 | 1.28 | 0.53 | -0.89 |
| Q3 | 2.65 | 1.08 | 0.07 | -0.62 |
| Q4 | 2.35 | 1.24 | 0.41 | -0.91 |
| Q5 | 2.77 | 1.26 | 0.15 | -0.89 |
| Q6 | 2.61 | 1.21 | 0.27 | -0.85 |
| Q7 | 1.91 | 1.14 | 1.06 | 0.04 |
| Q8 | 2.69 | 1.17 | -0.00 | -1.10 |
| Q9 | 2.36 | 1.30 | 0.26 | -0.96 |
| Q10 | 2.37 | 1.18 | 0.45 | -0.79 |
| Q11 | 2.16 | 1.13 | 0.64 | -0.66 |
| Q12 | 2.61 | 1.25 | 0.53 | -0.90 |

**Note:** Physical = QI, Q5, Q9; Verbal = Q2, Q6, Q10; Anger = Q3, Q7, Q11; Hostility = Q4, Q8, Q12

**Table S3: Inter-correlations (Pearson r)**

|  | **Mean** | **SD** | **Physical** | **Verbal** | **Anger** | **Hostility** |
| --- | --- | --- | --- | --- | --- | --- |
| Physical | 7.65 | 2.84 | 1.00 |  |  |  |
| Verbal | 7.34 | 2.50 | 0.62 | 1.00 |  |  |
| Anger | 6.72 | 2.50 | 0.55 | 0.60 | 1.00 |  |
| Hostility | 7.60 | 3.01 | 0.56 | 0.65 | 0.53 | 1.00 |

**Table S4: Factor-analysis checks & rotated loadings (exploratory)**

| **Item** | **F1** | **F2** | **F3** | **F4** | **Primary Factor** | **Max Loading** |
| --- | --- | --- | --- | --- | --- | --- |
| Q1 | 0.319 | -1.012 | -0.064 | 0.260 | Factor2 | 1.012 |
| Q2 | 0.244 | -0.404 | 0.129 | 0.797 | Factor4 | 0.797 |
| Q3 | 0.225 | -0.189 | 0.188 | 1.074 | Factor4 | 1.074 |
| Q4 | 0.376 | -0.873 | 0.265 | 0.300 | Factor2 | 0.873 |
| Q5 | 0.434 | -0.275 | 0.741 | 0.313 | Factor3 | 0.741 |
| Q6 | 0.560 | -0.262 | 0.643 | 0.174 | Factor3 | 0.643 |
| Q7 | 0.113 | 0.005 | 0.816 | 0.234 | Factor3 | 0.816 |
| Q8 | 0.108 | -0.768 | 0.446 | 0.125 | Factor2 | 0.768 |
| Q9 | 0.293 | -0.539 | 0.464 | 0.181 | Factor3 | 0.464 |
| Q10 | 0.897 | -0.494 | 0.282 | 0.022 | Factor1 | 0.897 |
| Q11 | 1.086 | -0.021 | 0.068 | 0.338 | Factor1 | 1.086 |
| Q12 | 0.964 | -0.534 | 0.208 | 0.129 | Factor1 | 0.964 |
